# Splenectomy for splenic metastases from malignant adrenal pheochromocytoma: a case report

**DOI:** 10.7497/j.issn.2095-3941.2013.02.009

**Published:** 2013-06

**Authors:** Xiao-Feng Duan

**Affiliations:** Department of Esophageal Cancer, Tianjin Medical University Cancer Institute and Hospital, Tianjin 300060, China

**Keywords:** Splenic metastasis, adrenal pheochromocytoma, splenectomy

## Abstract

Splenic metastasis is generally not a common clinical event. However, metastasis to the spleen from adrenal pheochromocytoma is extremely rare and has not been reported in literature. This report presents a case of a 58 year-old male patient who developed spleen-only metastases in July 2007. The patient had a previous history of left epinephroectomy for adrenal pheochromocytoma in January 2003. Abdominal computed tomography demonstrated multiple enhancing lesions suggestive of metastases; thus splenectomy was performed. Pathological examinations confirmed the diagnosis of splenic metastases from pheochromocytoma. The patient was alive without recurrence 48 months after splenectomy. This study is the first report on splenic metastasis from previous adrenal pheochromocytoma, and long-term survival was achieved by splenectomy. A history of malignancy indicates a high index of suspicion for splenic metastasis, and long-term survival can be achieved by splenectomy for spleen-only metastasis.

## Introduction

Primary and metastatic tumors of the spleen are described as unusual, excluding secondary involvement by lymphoma^1^. Given that metastatic carcinoma involving the spleen is usually a manifestation of widely disseminated disease, isolated splenic metastasis from malignancies is not a common occurrence. Recently, Sauer *et al.*^2^ reviewed the clinical rate of 29,364 patients with malignant tumors who developed metastases. They found that splenic involvement occurred in less than 1% of all metastases. Breast, lung, colorectal, and ovarian carcinomas and melanoma are the most common primary sources of splenic metastasis^3^. Splenic metastasis from pheochromocytoma has not yet been reported in the literature. This study presents an extremely rare case of a patient who underwent splenectomy for metastases originating from adrenal pheochromocytoma.

## Case report

This study was approved by the Cancer Hospital Ethic Committee and performed in accordance with the ethical standards of 2008 Declaration of Helsinki. Written informed consent was obtained from the patient.

In January 2003, a 54 year-old Chinese male patient underwent from left epinephroectomy for adrenal pheochromocytoma. Postoperative histological examination showed pheochromocytoma with adrenal capsule invasion. Given this information and associated increased risk of recurrence, he received a follow-up of abdominal ultrasonography every six months postoperatively and remained disease-free for the next 54 months.

**Figure 1:**
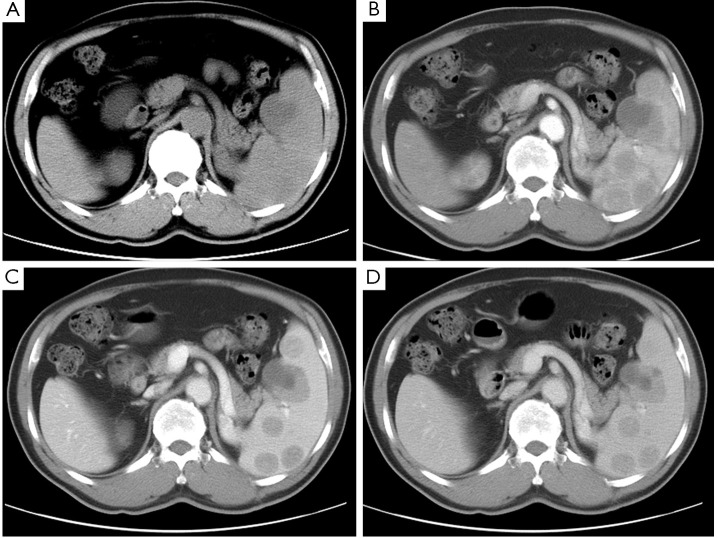
Abdominal computed tomography images of the patient. A. Plain scan; B. Contrast-enhanced scan at the arterial phase; C. Contrast-enhanced scan at the portal venous phase; D. Contrast-enhanced scan at the delayed phase.

In July 2007, splenic lesions were incidentally discovered via follow-up ultrasonography. Meanwhile, an increase in the fasting blood glucose (7.15 mmol/L) was also observed. The remainder of the laboratory tests, including liver and renal functions, blood cell counts, and epidemiological tests, was normal. Further abdominal plain computed tomography (CT) scans demonstrated an enlarged spleen with low-density and ill-defined lesions. Contrast-enhanced CT scans showed multiple moderately enhancing nodules that are suggestive of metastasis (**Figure 1**). No evidence of extra-splenic notable involvements was observed in a cross-sectional image of the chest and abdomen. After a multidisciplinary discussion, the splenic lesions were most likely thought to be splenic metastases from previous adrenal pheochromocytoma, and splenic resection was recommended. The patient underwent open splenectomy after a comprehensive assessment. He had an uncomplicated procedure/recovery and was discharged from the hospital on the seventh postoperative day.

The removed spleen macroscopically measured 15 cm × 11.5 cm × 6 cm. Serial slicing indicated grayish-white multiple nodules. The largest and smallest nodules measured 4.5 and 2 cm, respectively. Splenic metastases from pheochromocytoma were microscopically confirmed, and synaptophysin and chromogranin A were stained positive by immunohistochemistry. The tumor did not reach the splenic capsule, and no evidence of splenic hilar lymph node involvement was observed.

A post-splenectomy follow-up was performed every six months via abdominal ultrasonography or CT scan. The patient was still alive without recurrence 48 months after splenectomy on July 2011.

## Discussion

Pheochromocytoma usually originates from the adrenal medulla with pathological over-secretion of catecholamine, and only 10% of patients manifest malignant tumor^4^. Common distant spread sites of malignant pheochromocytoma include bone, liver, lung, and lymph nodes^5^. Spread to the spleen is extremely rare. To our knowledge, this report was the first to describe a patient who developed splenic metastases from adrenal pheochromocytoma after radical epinephroectomy.

Splenic metastasis is exceptionally rare. Several hypotheses have been proposed to explain the rarity of splenic metastasis^3^^,^^6^^-^^8^: (1) hemodynamic factors: the constant blood flow through the spleen, the sharp angle of the splenic artery branching from the celiac artery and the tortuosity of splenic artery, and the rhythmic contractions of the spleen all impede the implantation of cancer cells in the spleen; (2) physical barrier: the scarcity of lymphatic vessels in the spleen and the splenic capsule limit the transport of metastatic tumor cells into the spleen; (3) immunology: the presence of anti-tumor humoral factors and high concentration of phagocytes in the spleen have an inhibitory effect.

The wide and varied causes of splenic lesions present a diagnostic challenge. A splenic mass without any history of malignancy suggests a primary lesion, such as lymphoma, vascular tumors, or infectious lesions. By contrast, any past or present history of malignancy indicates a high index of suspicion for a splenic metastasis^9^. Splenic metastasis is most often incidentally detected via ultrasonography or CT in follow-up of cancer patients. Most cases are asymptomatic. The case in this report was asymptomatic and found via follow-up ultrasonography. CT scans were subsequently performed, and the results indicated the presence of splenic metastasis.

Splenic metastasis tends to have no clinical importance because it is often found in the context of disseminated malignancy. However, splenectomy is recommended in cases of isolated splenic metastases, which can achieve increased patient survival^2^. It can also be performed for palliative symptomatic relief, and reports show acceptable morbidity and improvements in quality of life^10^. Survival after splenectomy in patients with isolated splenic metastasis from pheochromocytoma is unknown. In this report, the patient acquired a long-term disease-free survival of over 48 months.

In conclusion, splenic metastasis in pheochromocytoma is extremely rare. It is incidentally detected on follow-up imaging findings without any symptoms. A long-term survival can be achieved by splenectomy in cases of spleen-only metastasis.
